# Sequence and phylogenetic analysis of nucleocapsid genes of porcine epidemic diarrhea virus (PEDV) strains in China

**DOI:** 10.1007/s00705-012-1592-4

**Published:** 2013-02-07

**Authors:** Zhili Li, Feng Chen, Yao Yuan, Xiduo Zeng, Zhongyan Wei, Ling Zhu, Baoli Sun, Qingmei Xie, Yongchang Cao, Chunyi Xue, Jingyun Ma, Yingzuo Bee

**Affiliations:** 1 College of Animal Science, South China Agricultural University, Tianhe District, Wushan Road, Guangzhou, 510642 Guangdong, People’s Republic of China; 2 State Key Laboratory of Biocontrol, College of Life Sciences, Sun Yat-sen University, Guangzhou, 510275 People’s Republic of China

## Abstract

Porcine epidemic diarrhea virus (PEDV) causes acute diarrhea and dehydration with high mortality rates in swine. It has become increasingly problematic in China. Since the nucleocapsid (N) protein is highly conserved, it is a candidate protein for early diagnosis and vaccine development. In this study, the N genes of 15 PEDV strains were amplified by RT-PCR and cloned into the pMT-19T vector, sequenced, and compared to each other as well as to PEDV reference strains. The nucleotide sequences of the N gene of the Chinese PEDV strains consist of 1326 nucleotides and encode a 441-aa-long peptide. The nucleotide sequences of the fifteen PEDV strains in our study were 96.1-100 % identical to each other, and the deduced amino acid sequences were 94.8-100 % identical. Sequence comparison with other PEDV strains selected from GenBank revealed that their nucleotide sequences were 94.2-99.7 % identical to those of the Chinese PEDV strains, and their deduced amino acid sequences were 94.1-99.5 % identical. In addition, the fifteen strains showed a high degree of nucleotide sequence identity to the early domestic strains (98.4-99.7 %) except the LZC strain, but less sequence identity to the vaccine strain (CV777) used in China (94.7-97.7 %). Phylogenetic analysis showed that the Chinese PEDV strains are composed of a separate cluster including three early domestic strains (JS-2004-02, LJB/03 and DX) but differ genetically from the vaccine strain (CV777) and the early Korean strains (Chinju99 and SM98).

## Introduction

Porcine epidemic diarrhea (PED), caused by porcine epidemic diarrhea virus (PEDV), is an acute, highly contagious, and devastating enteric disease that is characterized by severe enteritis and diarrhea, with high mortality rates in suckling pigs [1]. PED was first reported in England in 1971 [2]. Since then, PED occurs in most swine-raising countries in Europe, as well as China, Korea, Thailand, and Japan [3–6]. However, this disease is becoming a big concern especially in Asia, where outbreaks are often more acute and severe than those observed in Europe [7, 8]. Since the beginning of October 2010, a porcine epidemic diarrhea epizootic has been occurring in China, affecting pigs of all ages but characterized by high mortality rates among suckling piglets. The outbreak has been prevalent nationwide and has caused huge economic losses [9–11]. Most of the affected farms have lost 100 % of their newborn piglets, usually within 7 days, but sometimes even within only a few hours of birth. Few sows or boars show any clinical signs, which is inconsistent with a previous report of an outbreak in Thailand in 2007 that was characterized by pigs of all ages being infected and showing different degrees of diarrhea and anorexia [4]. In addition, in China, sporadic outbreaks of PED have been seen year round, and not just in the winter months.

Porcine epidemic diarrhea virus (PEDV) is an enveloped, single-stranded RNA virus, belonging to the order *Nidovirales*, family *Coronaviridae*, genus *Alphacoronavirus* [12–14]. The genome is comprised of a 5’ untranslated region (UTR), a 3’ UTR, and at least seven open reading frames (ORFs) that encode four structural proteins, (spike [S], envelope [E], membrane [M], and nucleocapsid [N]) and three non-structural proteins (replicase 1a and 1b and ORF3) [15–17]. It has been reported that the N protein binds to viral RNA, providing a structural basis for the helical nucleocapsid, which is a basic phosphoprotein associated with the genome [18]. In addition, the N protein is thought to be important in inducing cell-mediated immunity in the host [19]. The N protein participates in transcription of the viral genome, the formation of the viral core, and packaging of viral RNA [20, 21]. In the early stages of PEDV infection, a pig can produce high levels of antibodies against the N protein. Since the N protein is highly conserved, it is the best candidate protein for use as an antigen for early diagnosis reagents and vaccine development [22–24].

The purpose of the present study was to investigate the genetic characteristics of the N gene between 2010 and 2012 during PED outbreaks in different region of China. In this study, RNA was extracted directly from the feces or intestinal contents of piglets infected with PEDV. The N genes were cloned and sequenced, and the sequences were submitted to GenBank and compared with the N genes of other PEDV strains. In addition, the N protein motifs (including phosphorylation sites and hydrophilic regions) were identified. These data provide additional molecular epidemiological information on PEDV circulating in China and provide a basis for further development of diagnostic reagents and methods as well as assisting in vaccine selection.

## Materials and methods

### Sample collection

Porcine samples (including feces and intestinal contents) from piglets with severe watery diarrhea, dehydration and high mortality were collected from 55 farms in five provinces in China during February 2010 to March 2012. These samples were confirmed to be positive for PEDV by reverse transcription polymerase chain reaction (RT- PCR) [9].

### Primer design for RT- PCR

In order to determine the sequences of the N gene, primers were designed based on known published sequences in GenBank (CV777 and Brl/87). The sense primer was 5’- TGCGGTTCTCACAGATAGTG-3’, and the antisense primer was 5’-AAGTCGCTAGAAAAACACTCAGTAAT-3’. The size of the amplified product was predicted to be 1380 bp.

### RT-PCR

Viral RNA was extracted from samples using TRIzol Reagent (Invitrogen, CA, USA), resuspended in nuclease-free water, and kept at −70 °C until further use. Reverse transcription was performed at 50 °C for 30 min in a reaction mixture consisting of 2.5 μl RNA (0.2 μg), 1 μl primer (10 pmol), 1 μl Prime Script One Step Enzyme mix, 8 μl RNase-free H_2_O, and 12.5 μl 2 × One Step Buffer. The cycling conditions for the PCR were 94 °C for 2 min, followed by 32 cycles of denaturation (94 °C for 10 s), annealing (58 °C for 30 s), and extension (72 °C for 1 min), followed by a final extension at 72 °C for 7 min. Both the reverse transcription and the polymerase chain reaction were conducted using a PrimeScript One Step RT-PCR Kit (TaKaRa, Japan).

### Cloning and sequencing

The amplified PCR products were subjected to gel electrophoresis, excised from the agarose gel and purified using an Agarose Gel DNA Purification Kit (TaKaRa, Japan). The PCR products were cloned into the pMD19-T vector according to the manufacturer’s instructions (TaKaRa, Japan), three clones were sent to Shanghai Sangon Bioengineering Ltd. to be sequenced in both directions for each fragment, and the sequence was analyzed.

### Genome and amino acid analysis

Fifteen sequences were selected randomly and aligned using ClustalX software (version 1.83), Bioedit and DNASTAR to examine their genetic diversity. Phylogenetic trees were constructed by the neighbour-joining (NJ) method using Molecular Evolutionary Genetics Analysis (MEGA) software (version 4.0). Bootstrap values were estimated for 1000 replicates. The reference strains used for sequence alignment, sequence analysis, and phylogenetic analysis with the Chinese PEDV strains are shown in Table 1.

**Table 1:** Details of the strains and the reference strains used in this study

Strain	Geographic origin	Accession no.	Strain	Geographic origin	Accession no.
LJB/03	Heilongjiang, China, 2006	DQ072726	CH-JKA1-2011	Sanshui, Guangdong Province, China, 2011	JN173296
JS-2004-2	Jiangsu, China, 2004	AY653206	CH-QY1-2011	Qingyuan, Guangdong Province, China, 2011	JN173297
LZC	Gansu, China, 2006	EF185992	CH-QY2-2011	Qingyuan, Guangdong Province, China, 2011	JN173298
DX	Gansu, China, 2007	EU031893	CH-SWK1-2011	Xinfeng, Jiangxi Province, China, 2011	JN173300
Chinju99	Korean, 2002	AF237764	CH-SWK6-2011	Xinfeng, Jiangxi Province, China, 2011	JN173301
SM98	Korean, 2010	GU937797	CH-GD1-2010	Yunfu, Guangdong Province, China, 2010	JN173277
CV777	England, 2001	AF353511	CH-GD2-2010	Yunfu, Guangdong Province, China, 2010	JN173278
CH-GDXS4-2010	Foshan, Guangdong Province, China, 2010	JN173285	CH-SIC1-2011	Deyang, Sichuan Province, China, 2011	JQ390539
CH-GX1-2011	Guilin, Guangxi Province, China, 2011	JN173302	CH-SIC2-2011	Deyang, Sichuan Province, China, 2011	JQ390540
CH-GX2-2011	Guilin, Guangxi Province, China, 2011	JN173303	CH-JS1-2011	Huaian, Jiangsu Province, China, 2011	JQ390543
CH-GDHSY-2011	Yangjiang, Guangdong Province, China, 2011	JN173275	CH- JS2-2011	Huaian, Jiangsu Province, China, 2011	JQ390544

### Protein sequence analysis

The hydrophilic regions of the deduced amino acid sequences were analysed using DNASTAR software. The phosphorylation sites were predicted using NetPhis and NetPhos K analysis tools available at http://www.cbs.dtu.dk/services/NetPhos/ and http://www.cbs.dtu.dk/services/NetPhosK/.

## Results

### Homology analysis of the N gene

Sequence homology results were based on the fifteen Chinese PEDV strains and seven commonly used strains published in GenBank (Table 1). The fifteen Chinese field PEDV strains had 96.1-100 % nucleotide and 94.8-100 % deduced amino acid sequence identity to each other. Sequence comparison with the other seven selected strains of PEDV revealed that the Chinese field PEDV strains had nucleotide sequence identities of 94.2-99.7 % and deduced amino acid sequence identities of 94.1-99.5 %. In addition, the fifteen strains showed a high degree of nucleotide sequence identity to the early domestic strains (98.4-99.7 %) except the LZC strain, but less identity to the vaccine strain (CV777) used in China (94.7-97.7 %).

### Sequence analysis of the N gene

Sequence analysis revealed that none of the fifteen strains had insertions or deletions in the N gene. In each case, the N gene had an ORF of 1326 nucleotides coding for a 441-amino-acid protein. The alignment with the Chinju99 strain indicates that the N gene is highly conserved except for some point mutations located at amino acid positions 84, 123, 142, 144, 206, 242 to 255 292, 317, 350, 369 and 394 to 413 (shown in Table 2). The Chinese strains had 15 to 18 amino acids mismatched when compared to Chinju99. Interestingly, the sequences of the fifteen strains were highly conserved in the 5’ region (bases 1 to 252), while some variation was observed in the 3’ region (bases 1050 to 1233). This finding indicates that the N-terminal part of the protein is more conserved than the C-terminal part. In addition, there were four highly conserved regions at bp 1-83, 517-616, 950-1020 and 1106-1178.

**Table 2:** The amino acid point mutations of the Chinese strains compared to Chinju99

Strains	Positions of amino acid point mutations																	
	84	123	142	144	206	242	246	249	252	255	292	317	350	369	394	397	400	413
Chinju99	G	K	A	V	S	H	H	Q	K	N	R	P	I	A	I	Q	E	V
CH-SWK1-2011	G→A	K→N	A→T	V→A	S→N	H→L	H→Q	Q→K	K→R	N→S	R→A	P→S	I→T	A→S	I→A	Q→L	-	V→A
CH-SWK6-2011	G→A	K→N	A→T	V→A	S→N	H→L	H→Q	Q→K	K→R	N→S	R→A	P→S	I→T	A→S	I→A	Q→L	-	V→A
CH-QY1-2011	G→A	K→N	A→T	V→A	S→N	H→L	H→Q	Q→K	-	-	R→A	P→S	I→T	A→S	I→T	Q→L	E→D	-
CH-QY2-2011	G→A	K→N	A→T	V→A	S→N	H→L	H→Q	Q→K	-	-	R→A	P→S	I→T	A→S	I→T	Q→L	E→D	-
CH-JKA1-2011	G→A	-	A→T	V→S	S→N	H→L	H→Q	Q→K	-	-	R→A	P→S	I→T	A→S	I→T	Q→L	E→D	-
CH-GDXS4-2010	G→A	K→N	A→T	V→A	S→N	H→L	H→L	Q→K	K→R	N→S	R→A	P→S	I→T	A→S	I→A	Q→L	-	V→A
CH-GDHSY-2011	G→A	-	A→T	V→S	S→N	H→L	H→Q	Q→K	-	-	R→A	P→S	I→T	A→S	I→T	Q→L	E→D	-
CH-SIC1-2011	G→A	K→N	A→T	V→A	S→N	H→L	H→Q	Q→K	K→R	N→S	R→A	P→S	I→T	A→S	I→A	Q→L	-	V→A
CH-SIC2-2011	G→A	K→N	A→T	V→A	S→N	H→L	H→Q	K	K→R	N→S	R→A	P→S	I→T	A→S	I→A	Q→L	-	V→A
CH-GD1-2010	G→A	K→N	A→T	V→A	S→N	H→L	H→Q	Q→K	K→R	N→S	R→A	P→S	I→T	A→S	I→A	Q→L	-	V→A
CH-GD2-2010	G→A	-	A→T	V→S	S→N	H→L	H→Q	Q→K	-	-	R→A	P→S	I→T	A→S	I→T	Q→L	E→D	-
CH-GX1-2011	G→A	-	A→T	V→A	S→N	H→L	H→Q	Q→K	-	-	R→A	P→S	I→T	A→S	I→T	Q→L	E→D	-
CH-GX2-2011	G→A	-	A→T	V→A	S→N	H→L	H→Q	Q→K	-	-	R→A	P→S	I→T	A→S	I→T	Q→L	E→D	-
CH-JS1-2011	G→A	K→N	A→T	V→A	S→N	H→L	H→Q	Q→K	K→R	N→S	R→A	P→S	I→T	A→S	I→T	Q→L	-	-
CH- JS2-2011	G→A	K→N	A→T	V→A	S→N	H→L	H→Q	Q→K	K→R	N→S	R→A	P→S	I→T	A→S	I→T	Q→L	-	-

### Prediction of phosphorylation sites and analysis of hydrophilic regions of the N protein

**Fig. 1:**
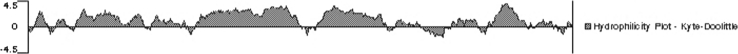
Hydrophilic regions in the central region of the N protein of the CH-GX1-2011 strain

The deduced amino sequence of the N gene of strain CH-GX1-2011 was randomly selected to be analyzed for specific motifs. The analysis indicated that the protein had seven potential asparagine (N)-linked glycosylation sites, consistent with the number seen in the Chinju99, LJB/03 and DX strains. Moreover, the CH-GX1-2011 strain had seven potential protein kinase C phosphorylation sites, nine casein kinase II phosphorylation sites, one tyrosine kinase phosphorylation site, and two cAMP- and cGMP-dependent protein kinase phosphorylation sites. In addition, a large hydrophilic region was identified in the central region of the protein (Fig. 1).

### Phylogenetic analysis of the N gene

**Fig. 2:**
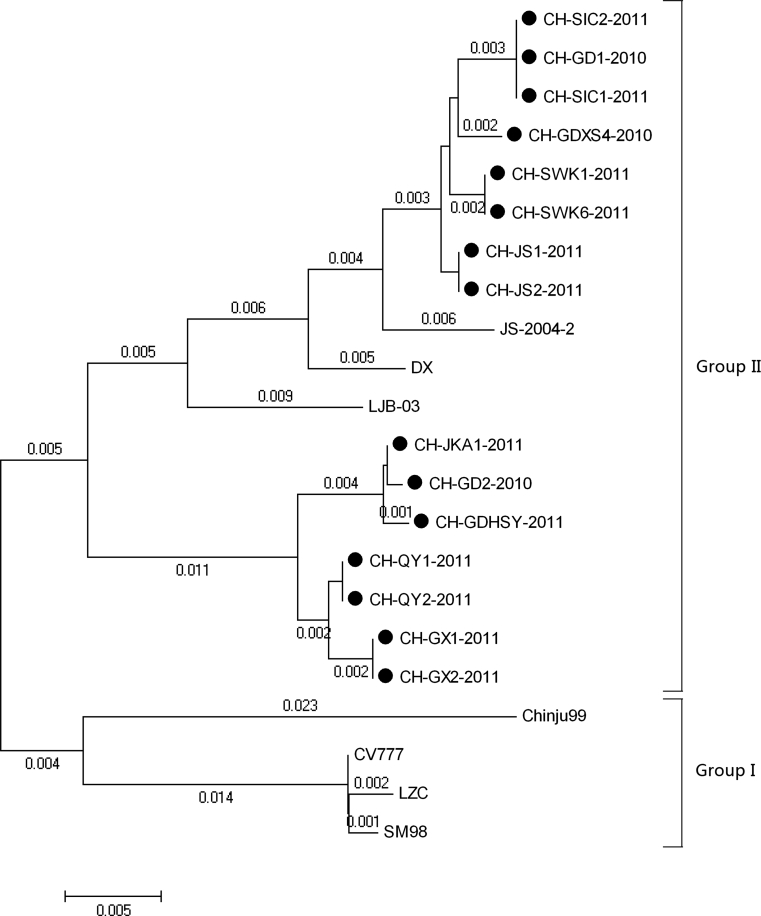
Phylogenetic analysis of the nucleotide sequences of the complete N gene of fifteen PEDV strains and PEDV reference strains. The tree was constructed based on the minimum-evolution method using MEGA4 software. Numbers above the branches indicate bootstrap values calculated from 1,000 bootstrap replicates. The strains used in our manuscript are marked by filled circles.. Accession numbers for PEDV reference strains used in the analysis are as follows: LJB/03 (China,2006;DQ072726), S-2004-2 (China,2004;AY653206), LZC(China,2006;EF185992), DX (China,2007;EU031893), Chinju99 (Korean,2002;AF237764), SM98 (Korean,2010;GU937797), CV777 (England,2001;AF353511)

Phylogenetic analysis based on nucleotide and encoded amino acid sequences of the N gene confirmed that all field strains fell into two groups (Fig. 2). Group I consisted of Chinju99 (Korean), CV777 (Europe), LZC (China), and SM98 (Korean), while group II contained 18 Chinese strains. The Chinese PEDV strains had a close relationship to the three domestic strains (JS-2004-02, LJB/03 and DX) and clustered together.

## Discussion

In the present study, the N genes of Chinese PEDV field strains isolated between 2010 and 2012 were amplified by RT-PCR, cloned and sequenced to determine the genetic characteristics of viruses causing PED outbreaks in China. The results confirmed that the N gene had an ORF of 1326 nucleotides, coding for a protein of 441 amino acids. None of the Chinese strains were found to have sequence insertions or deletions in their N genes. Sequence comparison with other PEDV strains selected from GenBank indicated that the N genes of the Chinese strains were highly conserved, even though these strains originated from different geographic regions. The alignment also showed that the N gene sequences have a high degree of nucleotide sequence identity. This could be useful information for the development of genetically engineered N proteins for vaccine development and prevention of PEDV infections.

The N protein is a phosphorylated structural protein that is associated with the viral genome and is abundant in virus-infected cells [25]. Therefore, the appearance of the N protein indicates replication of PEDV, and this can be used for early and accurate detection of virus replication in infected cells [23, 26]. Previous studies have shown that the N protein of the Chinju99 isolate has seven potential T- or S-linked phosphorylation sites [27]. In this study it was revealed that the CH-GX1-2011 strain (representing all of the 15 Chinese strains) had the same number of T- or S-linked phosphorylation sites as Chinju99 despite that fact that there were 15-18 mismatched amino acids when compared to Chinju99, LJB/03 and DX [28, 29]. Moreover, the CH-GX1-2011 strain had seven potential protein kinase C phosphorylation sites, nine casein kinase II phosphorylation sites, one tyrosine kinase phosphorylation site, and two cAMP- and cGMP-dependent protein kinase phosphorylation sites. There are larger hydrophilic regions in the center of the N protein that might play a role in transcription and replication of the viral genome.

Phylogenetic trees were constructed and analyzed using nucleotide and deduced amino acid sequences of the N gene. Similarities and differences among the PEDV strains were observed, which helped in elucidating the phylogenetic relationship between the Chinese PEDV strains and the reference PEDV strains. The results showed that the Chinese PEDV strains characterized in this study make up a separate cluster that includes three other Chinese strains (JS-2004-02, LJB/03 and DX), while they differed genetically from the vaccine strain (CV777) and the early Korean strains (Chinju99 and SM98).

In conclusion, the N genes of Chinese PEDV strains isolated in 2010-2012 during PED outbreaks were sequenced and compared to other reference strains. The N genes were found to be closely related to the N gene of earlier domestic PEDV isolates (JS-2004-02, LJB/03 and DX). The N gene was highly conserved but still had some unique point mutations as well as conserved regions. It is hoped that these data may add to other molecular epidemiological studies of PEDV in China and neighboring countries. Furthermore, it may also lay the foundation for further development and selection of PEDV vaccines.
